# Optical molecular imaging technology and its application in precise surgical navigation of liver cancer

**DOI:** 10.7150/thno.102671

**Published:** 2025-01-01

**Authors:** Pan He, Haitian Tang, Yating Zheng, Xiao Xu, Xuqi Peng, Tao Jiang, Yongfu Xiong, Yang Zhang, Yu Zhang, Gang Liu

**Affiliations:** 1 Department of Hepatobiliary and Pancreas Surgery, Sichuan Provincial People's Hospital, University of Electronic Science and Technology of China, Chengdu 611731, China.; 2 State Key Laboratory of Vaccines for Infectious Diseases, Center for Molecular Imaging and Translational Medicine, Xiang An Biomedicine Laboratory, National Innovation Platform for Industry-Education Integration in Vaccine Research, School of Public Health, Xiamen University, Xiamen 361002, China.; 3 Department of General Surgery, Institute of Hepatobiliary-Pancreatic-Intestinal Diseases, Affiliated Hospital of North Sichuan Medical College, Nanchong 637000, China.

**Keywords:** Molecular imaging, Theranostics, Liver cancer, Surgical navigation

## Abstract

Recent innovations in medical imaging technology have placed molecular imaging techniques at the forefront of diagnostic advancements. The current research trajectory in this field aims to integrate personalized molecular data of patients and diseases with traditional anatomical imaging data, enabling more precise, non-invasive, or minimally invasive diagnostic options for clinical medicine. This article provides an in-depth exploration of the basic principles and system components of optical molecular imaging technology. It also examines commonly used targeting mechanisms of optical probes, focusing especially on indocyanine green—the FDA-approved optical dye widely used in clinical settings—and its specific applications in diagnosing and treating liver cancer. Finally, this review highlights the advantages, limitations, and future challenges facing optical molecular imaging technology, offering a comprehensive overview of recent advances, clinical applications, and potential impacts on liver cancer treatment strategies.

## Introduction

Advancements in imaging technologies have significantly accelerated the development of molecular imaging, reshaping diagnostic and treatment paradigms in clinical medicine 1,2. Molecular imaging uses two- and three-dimensional imaging devices to capture and differentiate various physiological and pathological processes at the cellular and even subcellular levels *in vivo*. This approach enables qualitative and quantitative analyses, providing highly sensitive detection methods to facilitate early disease diagnosis 3. Unlike traditional imaging, which primarily visualizes structural abnormalities, molecular imaging leverages biochemical and intracellular pathways to illuminate disease initiation and progression mechanisms 4. As research into molecular disease mechanisms has deepened, the potential of molecular imaging to enhance clinical understanding and treatment strategies has advanced, driving its rapid integration into both foundational and clinical disease research.

In clinical practice, molecular imaging is increasingly valued for its ability to assess cellular-level changes that often precede detectable anatomical alterations, thus aiding in early diagnosis and disease characterization 5-7. This approach is particularly adept at identifying functional changes within tissues, enhancing sensitivity over traditional imaging modalities. Additionally, molecular imaging has proven invaluable for early-stage drug evaluation, allowing researchers to precisely and quantitatively monitor the effects of drugs on molecular targets *in vivo* 8. In gene therapy, molecular imaging facilitates the detection of genetic-level changes, enabling the real-time monitoring of disease onset and progression before phenotypic alterations become evident.

**Figure 1:**
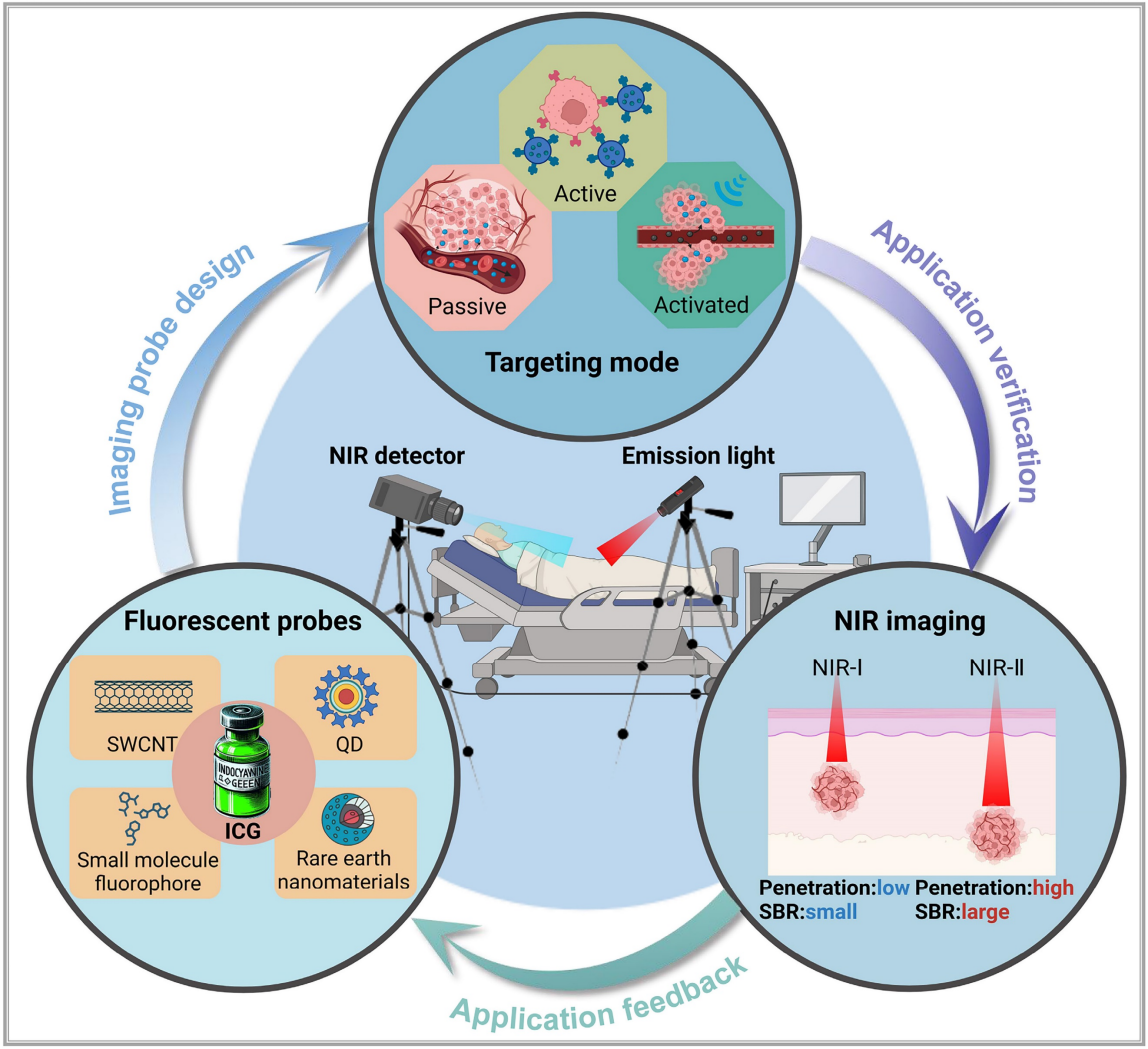
An illustration that represents optical molecular imaging techniques and their key applications in precision surgery navigation, including common fluorescent probes, an introduction to the targeting methods of probes, and a comparison of NIR-I and NIR-II imaging. SWCNT = Single-walled carbon nano tube, QD = Quantum dot. This figure was created with Biorender.com.

In summary, molecular imaging is an interdisciplinary field encompassing various imaging modalities, such as positron emission tomography and single-photon emission computed tomography, both rooted in nuclear medicine, as well as imaging based on nuclear magnetic resonance and optical molecular imaging techniques 9. In recent years, optical molecular imaging has emerged as a promising modality for cancer diagnosis and treatment, particularly in enhancing precision surgery. However, the complexity of visualizing anatomical structures and physiological functions in this modality presents challenges in designing high-quality imaging systems and developing targeted probes, which impacts the clinical translation of applications 10. Based on current optical imaging systems and strategies 11, this review (**Figure 1**) delves into the fundamental principles and components of optical molecular imaging. Furthermore, it examines common targeting mechanisms of optical probes, emphasizing indocyanine green (ICG), a Food and Drug Administration (FDA)-approved optical dye for clinical applications, specifically for diagnosing and treating liver cancer. Lastly, the review discusses the benefits, limitations, and future challenges of optical molecular imaging.

## Optical molecular imaging technology

Optical molecular imaging is a non-invasive, real-time, and high-resolution imaging modality that combines optical technology with biomolecular labeling. It detects light signals emitted by endogenous or exogenous agents in tissues and translates cellular activities into visual patterns through specialized optical imaging devices 12. The main components of an optical molecular imaging system include the luminescent signal source, light source transmission and positioning systems, and the optical signal reception and analysis systems. The following detailed review provides an overview of these components.

### Luminous signal source of the imaging system

Optical molecular imaging systems rely on diverse luminescent signal sources with significantly varying biological and optical characteristics, requiring careful selection based on specific applications 13. In general, luminescent sources in optical imaging systems fall into two categories: endogenous and exogenous signals. Endogenous signals are directly related to living organisms, such as electronic energy transitions generated by biochemical reactions within the body, energy transitions triggered by external radiation or internal biochemical processes 14, for example, organisms such as fireflies naturally produce luminescence from biochemical reactions 15. However, in humans, spontaneous luminescence is relatively weak and lacks contrast against the background, making it difficult to distinguish effectively and unsuitable as a light source for optical imaging technology. Additionally, this signal can interfere with imaging data, affecting the detection results of the imaging system. Therefore, specific measures are often required in optical molecular imaging to eliminate or suppress the inherent luminescence of tissues to obtain clearer imaging results. The second category involves exogenous signals produced by luminescent materials, such as fluorescent dyes 16, fluorescent proteins 17, and quantum dots 18, either through spontaneous emission or light stimulation.

In developing optical biological probes, the luminescent material is crucial for probe distribution and imaging contrast. Although numerous fluorescent materials are available, only a very few are FDA-approved for human use, including ICG, sodium fluorescein, pafolacanine, and methylene blue 19,20. Among them, ICG is widely recognized for its unique advantages in clinical imaging. Specifically, ICG's emission of near-infrared (NIR) light improves imaging contrast by operating outside the body's natural luminescence spectrum. Additionally, NIR light has lower absorption and scattering in tissues, allowing deeper tissue penetration 21,22. Beyond these benefits, ICG demonstrates exceptional utility for imaging in both the first and second NIR regions, making it particularly valuable in liver cancer imaging.

### Common targeting methods for optical molecular imaging

**Figure 2:**
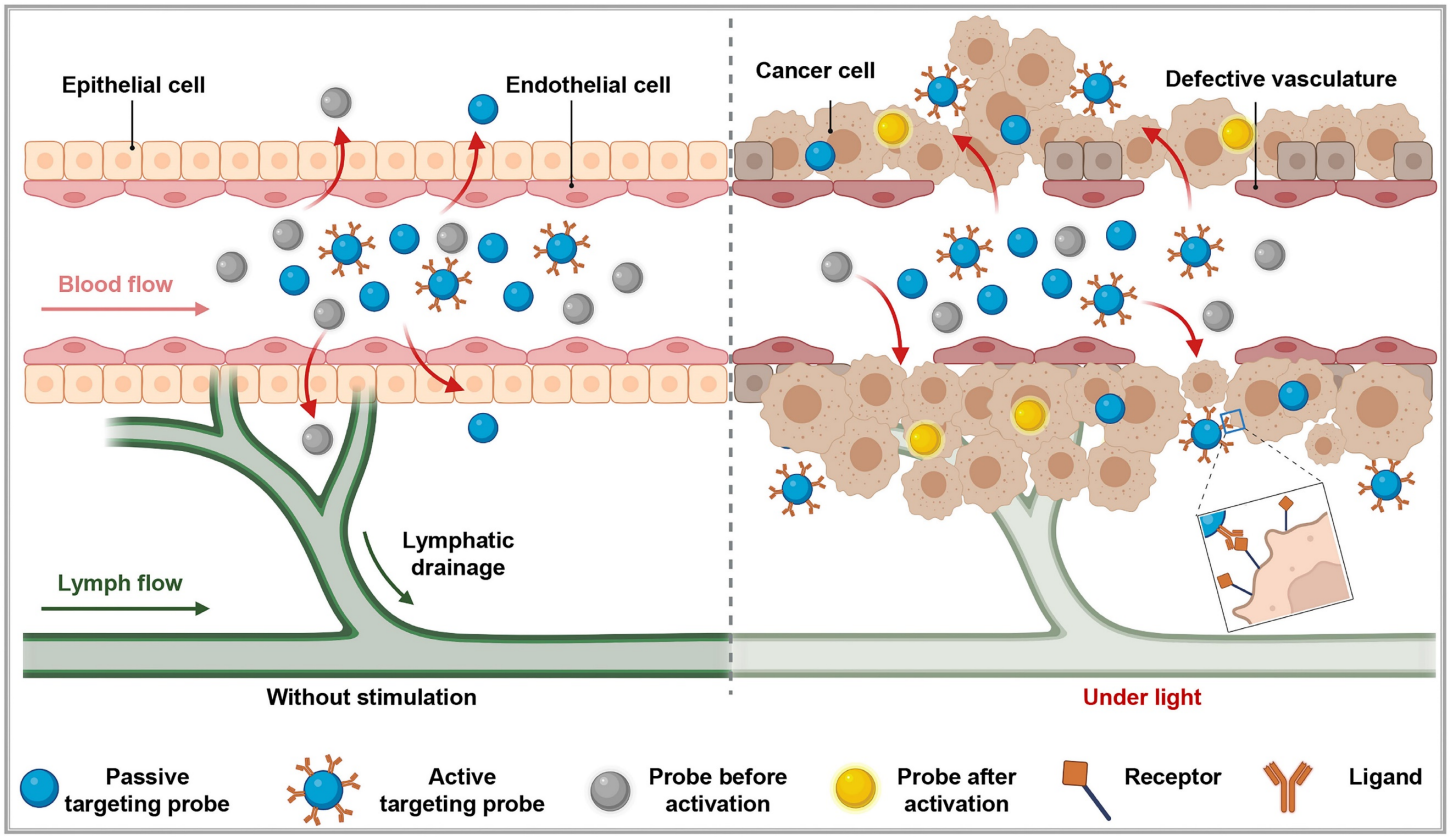
Three principal targeting strategies for nanoprobes: In the absence of an external excitation source, active targeting probes do not migrate from blood vessels into surrounding tissues. Some passive targeting and activatable probes can enter surrounding tissues via the enhanced permeability and retention effect. However, under these conditions, activatable probes lack drug activity (depicted as gray). When pathological changes, such as cancer, occur in the surrounding tissues, active targeting probes can specifically target and penetrate the diseased area. Meanwhile, activatable probes exhibit drug activity (depicted as yellow) under the influence of an external excitation source. This figure was created with Biorender.com.

To achieve precise imaging of the target areas, it is necessary to first accurately deliver luminescent materials to specific target points and then emit light signals. Depending on the use of carriers and their characteristics, the delivery of luminescent dyes to the target areas can be categorized into three: passive targeting, active targeting, and *in situ* activated probes (**Figure 2**).

#### Passive targeting

The most well-known theory in the passive targeting mechanism is the Enhanced Permeability and Retention (EPR) effect, which relies on the permeability of specific tissues or organs, guiding large molecular substances (such as nanoparticles and liposomes) to naturally accumulate in non-specific diseased tissues within the body. The mechanism of this effect can be attributed to two factors: (I) the disorganized structure and discontinuous endothelium of tumor neovasculature leading to excessive permeability of large molecules, and (II) the lack of an effective lymphatic drainage system in the tumor region, which causes accumulation of large molecular substances in that area 23,24. Fluorescent substances with passive targeting capabilities are generally composed of one or more luminescent materials to form a macromolecular contrast agent. After injection into the body, they do not bind to targeting ligands, rather directly enter the body, mainly in the blood. Therefore, regions with rich blood supply (vessels, high metabolic areas, and tumor neovasculature) appear as bright areas. This method is commonly employed in vascular and urinary tract imaging studies among others. Indeed, these macromolecules can also leak out into diseased tissues through incomplete or damaged vascular endothelial gaps, aiding visualization. ICG, for example, has been clinically applied in ophthalmic optical imaging technology for over 40 years through this approach 25. Moreover, experimental evidence has demonstrated that ICG achieves optical imaging of certain tumors through these non-specific means 19,20. Particularly, as ICG is exclusively cleared through the liver, it exhibits specific accumulation in liver cancer tissues, thereby providing a new method for the diagnosis and surgical treatment of liver cancer 26.

While the EPR effect demonstrates notable results in animal models, its effectiveness in clinical settings often falls short, raising questions related to its existence and prompting the exploration of new mechanisms to explain the passive targeting effects of nanomedicines. Sindhwanni 27 proposed that endocytosis might be the primary mechanism for the accumulation of nanoparticles at the tumor sites. They adopted the "Zombie" model to distinguish between passive penetration and active transport through endothelial cells, attempting to determine the dominant mechanism for nanoparticle accumulation in the tumor tissues. Simultaneously, Wang 28 suggested that the basement membrane around tumor blood vessels forms a barrier that hinders the penetration of nanodrugs through the tumor vascular wall. The authors demonstrated that the subendothelial space formed between the basement membrane surrounding tumor blood vessels and endothelial cells serves as a barrier where nanoparticles are intercepted, thereby forming a "subendothelial nanoparticle pool." This basement membrane barrier not only hinders the penetration of nanoparticles into deeper layers of tumor tissues but also restricts their ability to pass through the inter-endothelial gaps of endothelial cells. The synergistic immune-driven strategy induced by local hyperthermia overcomes this barrier and enhances the delivery of nanotherapies to tumors.

#### Active targeting

Active targeting probes in biomedicine are composed of highly specific ligands coupled with fluorescent markers, allowing for precise recognition and binding to target tissues. By combining fluorescent markers with targeting ligands, these biologically targeted probes offer advanced recognition capabilities, enabling specific imaging of molecular targets 29. Compared to passive targeting techniques, active targeting probes reflect the expression levels of target molecules more accurately by measuring fluorescent signal intensity. This high specificity minimizes interference from non-specific factors such as tumor microenvironment variability or vascular permeability changes 30. Active targeting ligands are diverse and not limited to antibodies 31; they also include small molecules 32, short peptide sequences 33, nucleic acid aptamers 34, proteins, and others 35,36. Each type of ligand has distinct advantages and limitations, allowing for tailored probe selection based on specific imaging requirements. However, the prolonged half-life of ligand-fluorescent probes can sometimes reduce image clarity, as background signals may compete with target signals 37. To enhance imaging contrast, two strategies can be employed: introducing a molecule after probe binding to the target, which binds to unbound probes in the bloodstream and promotes clearance, and applying fluorescence resonance energy transfer technology, where a molecule binds to the unbound probe, inducing fluorescence quenching to minimize non-specific signals 38.

**Figure 3:**
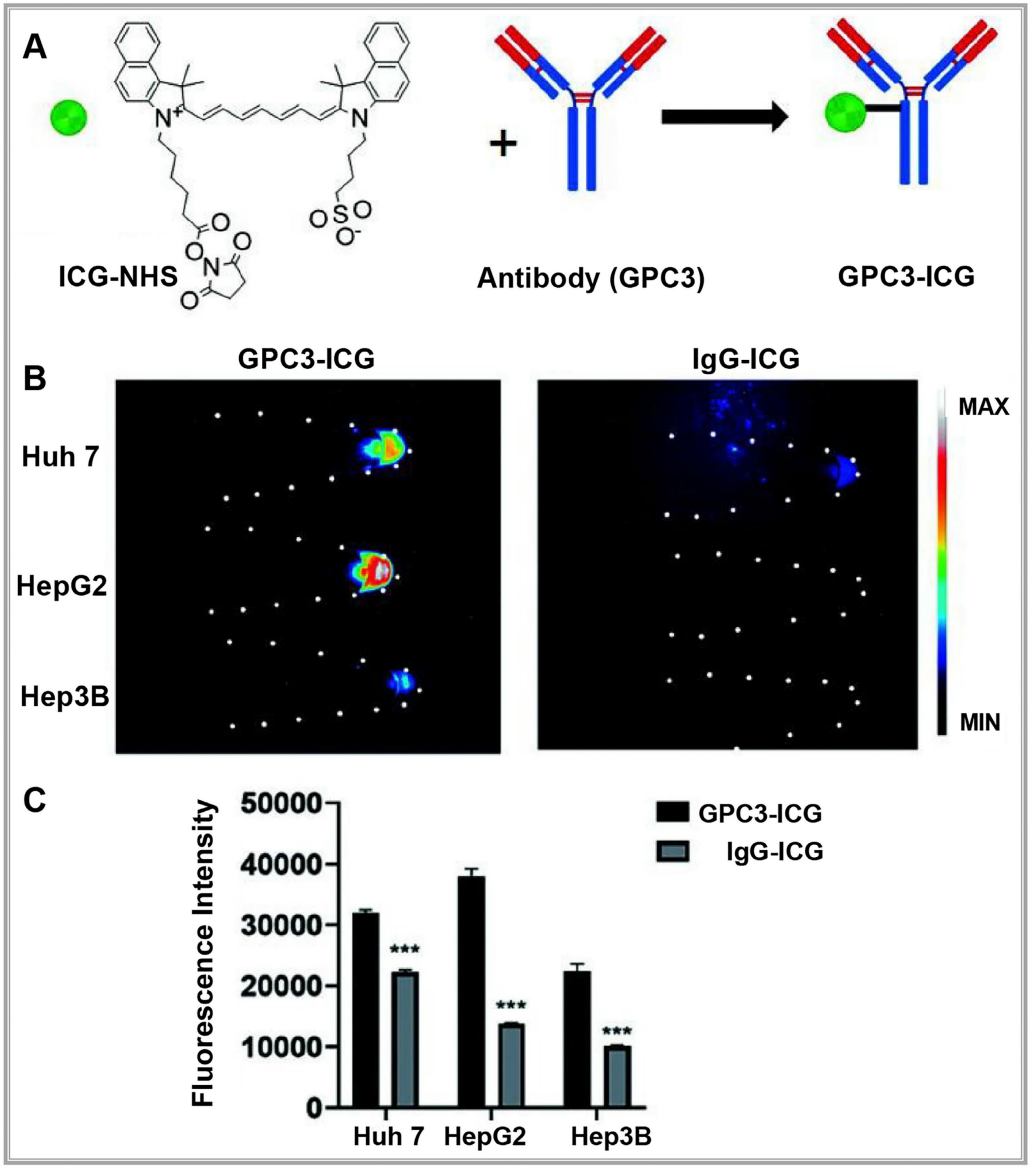
**(A)** NIR-II imaging of hepatocellular carcinoma based on a humanized anti-GPC3 antibody. **(B)** Different probes (left GPC3-ICG, right IgG-ICG) were incubated with Huh-7, Hep G2, and Hep 3B cell lines, and NIR-II images of the cell pellets were captured. **(C)** Quantification analysis of the NIR-II fluorescence intensity in different cell lines. The error bars indicate mean ± SD, **p < 0.01 with two-tailed Student's t-test. Reproduced with permission from 42, copyright 2022, Royal Society of Chemistry.

With the distinctive capabilities of nanomedicine in cancer diagnosis and treatment, researchers have developed actively targeted probes that attach to specific biomarkers by precisely modifying nanocarrier surfaces 39. These advancements allow for early disease diagnosis, real-time monitoring of disease progression, and assessment of therapeutic effectiveness. For example, the Bevacizumab-IRDye 800CW and Angiostamp800 probes, developed by Hurbin, provide superior tumor detection and are resistant to rapid clearance by the body 40. Swamy expanded on this approach by designing a π-conjugated ICG derivative paired with a monoclonal antibody to image human epidermal growth factor receptor 2- and epidermal growth factor receptor-positive breast cancers in patients, achieving promising results. Compared to traditional ICG, these specific fluorescent probes enable more accurate detection of tumors and metastatic sites, facilitating precise tumor resections 41. In another example, Shi labeled a humanized anti-GPC3 monoclonal antibody with ICG to create a NIR-II fluorescent probe (GPC3-ICG) designed to specifically target hepatocellular carcinoma (HCC) (**Figure 3A**). GPC3-ICG demonstrated high specificity and uptake in HCC cell lines (Huh-7, Hep G2, Hep 3B), exhibiting excellent efficiency and specificity (**Figure 3B-C**) 42.

#### Activating optical probe

Activatable optical probes rely on specific environmental triggers, such as variations in pH, enzyme presence, or light exposure, to activate and enable optical imaging 43. Typically inactive under normal conditions, these probes are designed to become active and emit signals only upon encountering specific conditions in the target area, providing higher contrast fluorescence imaging compared to "always-on" probes 44,45.

**Figure 4:**
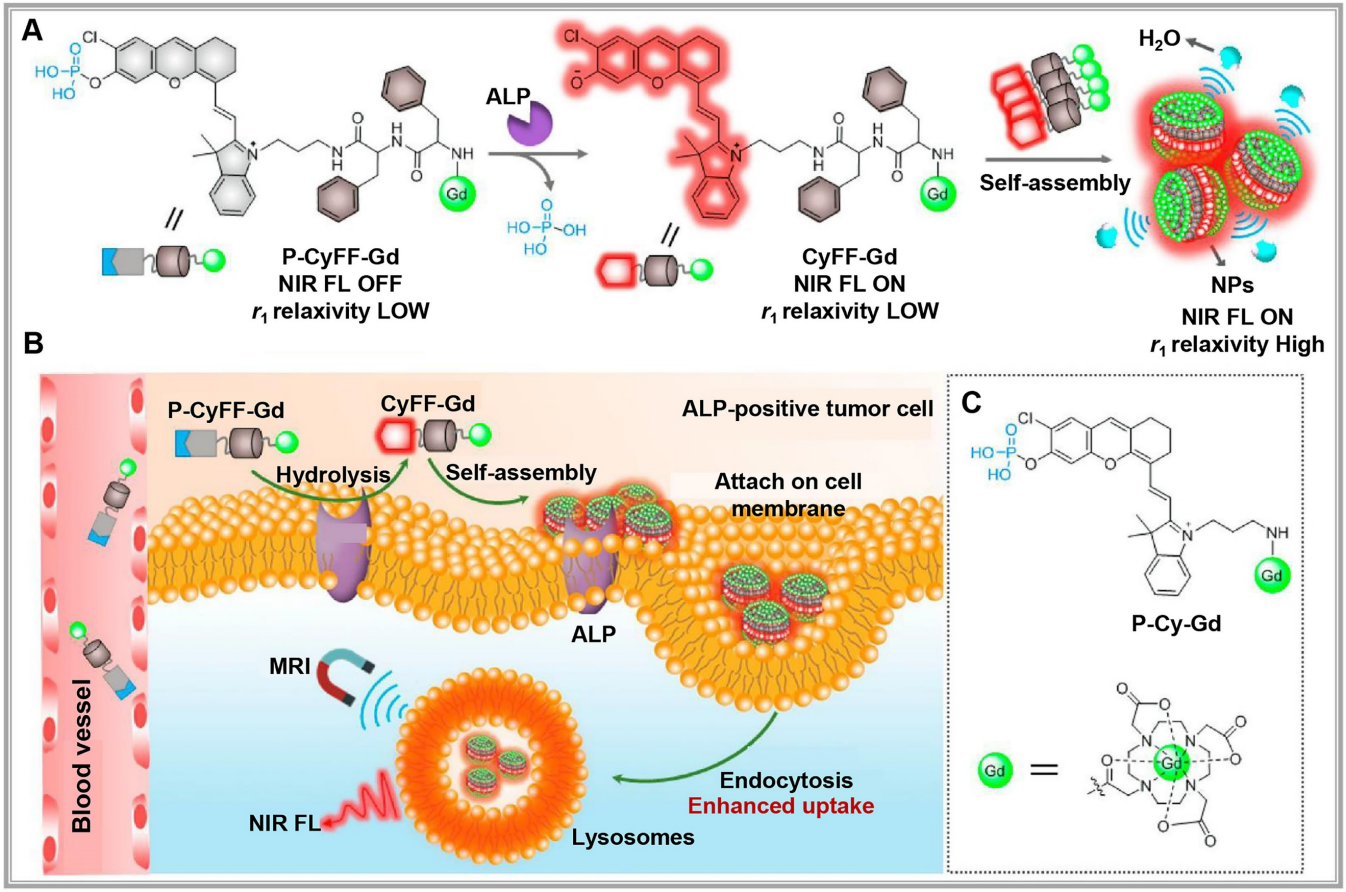
**(A)** Chemical structure of P-CyFF-Gd and proposed alkaline phosphatase (ALP)-mediated fluorogenic reaction and *in situ* self-assembly of P-CyFF-Gd into nanoparticles** (**NPs) that show increased NIR FL and r1 relaxivity. **(B)** Proposed mechanism of P-CyFF-Gd for NIR FL/MR bimodality imaging of ALP-positive tumor cells *in vivo*. Following systemic administration into mice, P-CyFF-Gd as a small molecule may easily across blood vessel and diffuse into tumor tissues. In tumor cells that express high levels of ALP, P-CyFF-Gd is dephosphorylated by membrane-bound ALP and converted into fluorescent CyFF-Gd, which subsequently selfassembles into fluorescent and magnetic NPs. **(C)** Chemical structure of the designed nonassembled control probe, P-Cy-Gd. Reproduced with permission from 51, copyright 2019, American Chemical Society.

Activatable optical probes fall into two main categories: activatable probes and binding probes. Activatable probes often consist of a fluorophore paired with a quencher, engineered to maintain proximity to precisely control fluorescence signal activation and quenching. For example, a peptide sequence linking the fluorophore and quencher can be tailored to respond to specific protease activities in the target region 46,47. By contrast, binding probes emit signals upon binding to specific biomarkers 37,48. For example, in DNA methylation assessment, probes targeting cytosine selectively bind to methylated alleles, emitting a fluorescent signal 49. Researchers have recently developed new "switch-like" probe strategies 50. For instance, alkaline phosphatase (ALP)-activated NIR fluorescence and magnetic resonance bimodal probes respond to ALP overexpression on cell membranes, forming nanoparticles visible through cryo-SEM imaging (**Figure 4A**). This bimodal approach enhances NIR fluorescence (>70-fold at 710 nm) and improves r1 relaxivity (~2.3-fold), allowing for real-time, high-sensitivity, high-resolution imaging and localization of ALP activity in live tumor cells and mice (**Figure 4B-C**) 51.

#### Combined targeting and multimodal imaging strategies

In clinical settings, combining targeted probe delivery with multimodal imaging can significantly increase drug delivery precision and drug concentration at target sites. For example, nano-targeted probes use specific antibodies to actively target lesions and accumulate passively in tumor tissue via the EPR effect, boosting local probe concentration 52. Additionally, optical probes combined with other imaging modalities can improve diagnostic accuracy and offer better therapeutic guidance, thus overcoming the limitations of single imaging methods. For instance, combining NIR dyes with gadolinium (Gd)-based nanoparticles enables multimodal MRI and NIR imaging 53,54. Furthermore, labeling NIR dyes with radioactive isotopes allows for the high sensitivity of PET, SPECT, and fluorescence imaging, facilitating early tumor diagnosis and treatment 55-57.

**Figure 5:**
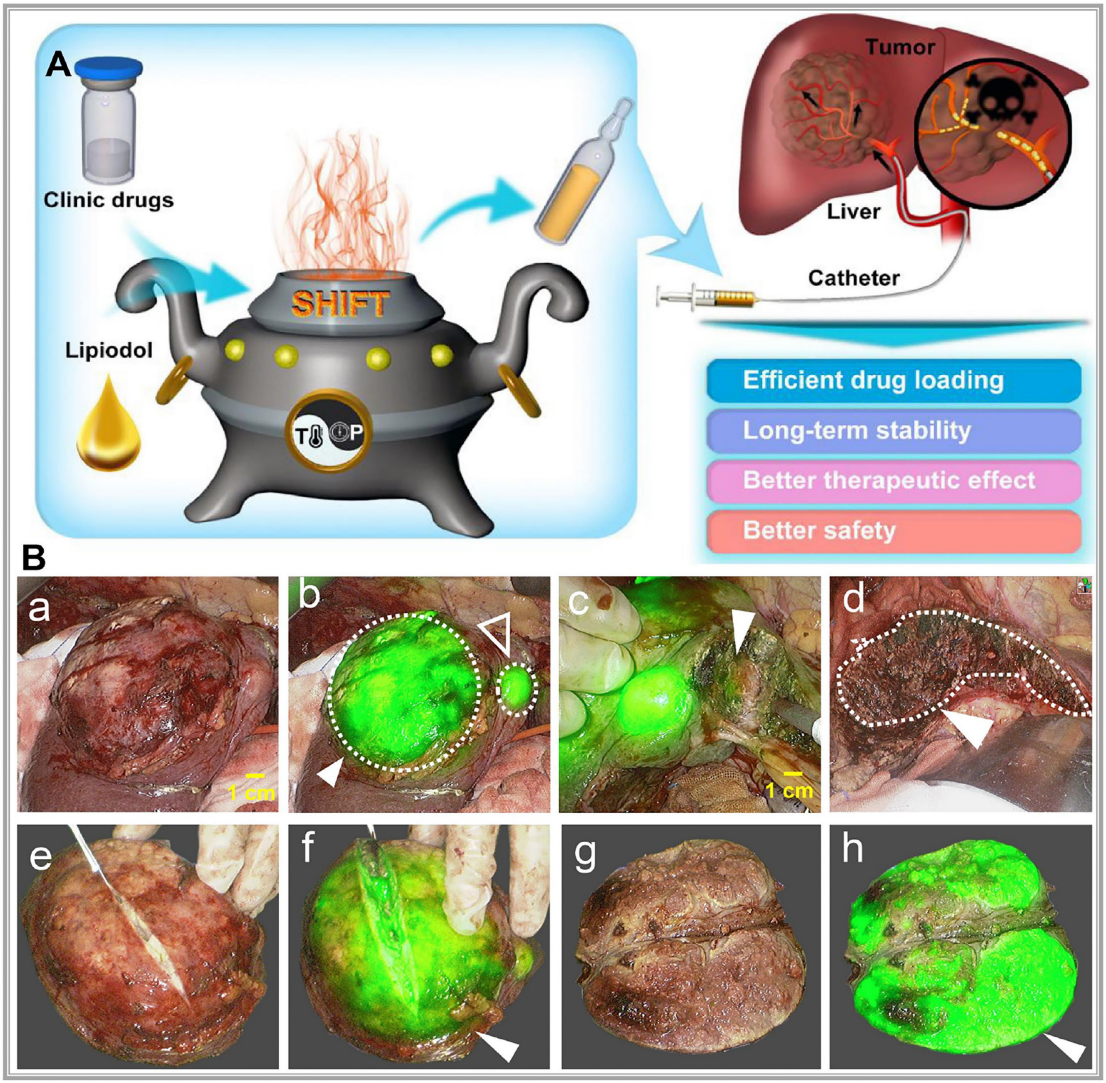
**(A)** Schematic illustration of superstable homogeneous intermixed formulation technology (SHIFT) as a revolutionary strategy for transhepatic arterial chem otherapy and embolization (TACE). Reproduced with permission from 59, copyright 2020, Elsevier. **(B)** The clinic drugs and lipiodol are introduced to develop formulations with SHIFT at a controlled temperature and pressure overcoming current challenges in the liver cancer treatment with TACE. Reproduced with permission from 60, copyright 2022, Wiley.

Precision-guided injections can also enhance targeting when combined with other imaging techniques. For example, ultrasound imaging provides real-time visualization of tissue structure, enabling the precise injection of optical probes into target areas for detailed molecular or cellular imaging 58. Alternatively, interventional techniques allow optical probes to be directly delivered to tumors via microcatheter superselection of tumor-feeding arteries, thereby increasing the local drug concentration in the tumor and improving imaging accuracy. Our research team has pioneered interventional optical molecular imaging, developing a super-stable homogeneous drug formulation technology that stably combines fluorescent dyes with interventional embolic agents (**Figure 5A**) 59. This innovation provides a promising approach for integrated diagnosis and treatment of liver cancer (**Figure 5B**) 60.

### Optical imaging system

NIR fluorescence imaging offers superior tissue penetration, low cost, high sensitivity, and safety advantages compared with visible light. These qualities make it an invaluable tool for real-time navigation during cancer surgeries 61. Achieving high-quality optical imaging relies on both a robust imaging system and effective fluorescent probes. Being key components of molecular imaging, fluorescence imaging systems capture light emitted by fluorophores excited by an external light source. This light is transformed by photoelectric conversion devices into digital signals, and then, displayed for the observer. Such systems typically include lenses, filters, electronic coupling devices, or complementary metal-oxide-semiconductor detectors, laser scanning or detection systems, lasers, computers, and biomedical signal processing software 19.

The goal of optical imaging systems is to capture and accurately display optical signals carrying specific biological information. Broadly, these systems are categorized into two main types based on their detection purposes: microscopic fluorescence imaging devices and macroscopic fluorescence imaging devices. However, significant technical and functional differences existed among devices based on different detection needs. With observation fields in the micrometer to sub-millimeter range, microscopic imaging devices provide high sensitivity and resolution for detailed visualization of tissue structures 62. Examples include multiphoton microscopes, single-photon microscopes, laser scanning confocal microscopes, spectral encoding endoscopes, fluorescence resonance energy transfer microscopes, and radiomolecular imaging devices. These are predominantly used for *in vitro* or *in vivo* microscopic analysis. Macroscopic fluorescence imaging devices, with observation fields typically in the centimeter to millimeter range, are designed for preclinical studies and clinical applications. Examples include fluorescence imaging and bioluminescence imaging devices, which use non-contact external detection or endoscopic detection for studies on small animals and humans. Compared to microscopic imaging devices, these have a simpler structure and are well-suited for clinical or and research purposes 63,64.

**Figure 6:**
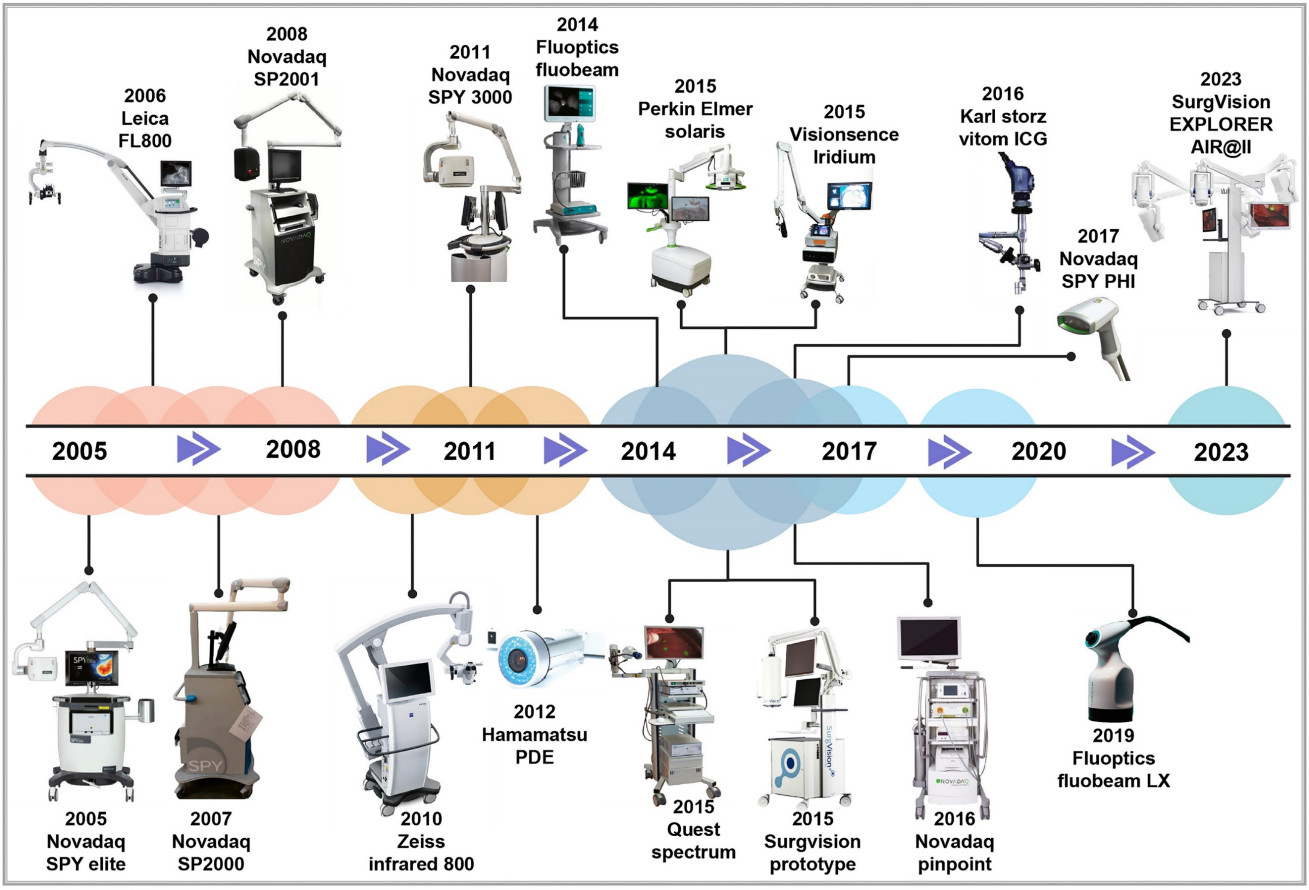
The developmental history of FDA-approved near-infrared fluorescence imaging and navigation systems. Reproduced with permission from 71-73. This figure was created with Biorender.com.

The origins of macroscopic fluorescence imaging date back to 1994, when Roger Y. Tsien improved green fluorescent protein technology. This breakthrough enabled the pairing of lasers with charge coupled device cameras, leading to the development of *in vivo* fluorescence imaging devices 65-67. Since then, global research institutions have advanced *in vivo* optical imaging devices to meet the growing demands of scientific research and clinical applications. For instance, in 1998, Professor Contag at Stanford University, in collaboration with partners, co-founded Xenogen Corporation, which pioneered the IVIS imaging system—a widely used bioluminescence imaging platform. In 2004, Professor Ge Wang's research team at Virginia Tech successfully developed an optical-CT multimodal imaging system, advancing the precision and scope of imaging technology. In 2005, Novadaq Technologies' SPY™ system became the first FDA-approved fluorescence molecular imaging system. This system is widely used in vascular surgery and for assisting surgical outcomes in breast cancer procedures 68. In 2009, the Molecular Imaging Research and Development Center at the Institute of Automation, Chinese Academy of Sciences developed an original bioluminescence imaging system with patents in both the U.S. and China. In 2019, Beijing Digital Precision Medical Technology Co., Ltd. expanded applications for intraoperative navigation, allowing real-time localization of small cancer lesions (< 5mm) 69. By 2022, the real-time super-sensitive fluorescence imaging system developed by the Tsinghua-IDG/McGovern Brain Institute team developed a high-sensitivity fluorescence imaging system, pushing the boundaries of imaging quality beyond particle noise limits, enhancing fluorescence imaging for biological research and clinical applications 70. As the concepts of minimally invasive and precision surgery keep on evolving, commercially approved imaging and navigation devices have become integral in surgical guidance. Common clinical imaging and navigation devices used for clinical diagnosis and treatment are presented in **Figure 6**.

### Clinical application of optical molecular imaging technology

Applications of optical molecular imaging technology can be categorized into three primary areas: (1) *In vivo* functional molecular detection: This approach uses *ex vivo* two- or three-dimensional optical imaging devices to analyze molecular functions and quantify molecules in living tumor tissues or diseased areas. (2) Therapeutics: In this category, multimodal probes are introduced into the body to not only image tumor tissues optically but also deliver therapeutic agents. (3) Intraoperative precision navigation: Optical probes are injected into the body before surgery. Specific lasers from the imaging system stimulate the probes to emit detectable signals. These signals outline detailed morphological information of the lesion on-screen, aiding surgeons in performing highly accurate procedures. This application has gained widespread use, particularly in hepatobiliary surgery, as fluorescence imaging offers high sensitivity and specificity, making it a valuable tool in diagnosing and treating liver cancer 74. Fluorescent probes can selectively bind to liver cancer cells or specific molecules in the tumor microenvironment, emitting fluorescence signals that allow visualization of the cancerous areas. This method helps doctors identify the location and quantity of cancerous lesions with higher precision. It also provides real-time guidance for lesion removal during surgery, enhancing the accuracy and completeness of the resection 75. These advancements significantly reduce the risk of postoperative recurrence and improve long-term survival rates for liver cancer patients.

Despite these advancements, the effectiveness of fluorescence imaging for liver cancer depends on stable and reliable fluorescence probes. Currently, available surgical navigation probes for liver cancer are limited, with intraoperative navigation primarily relying on ICG. However, ICG's low specificity and limited tissue penetration present challenges. Ideally, fluorescence probes for liver cancer imaging should have the following features: (1) ability to detect low concentrations of target molecules, enabling precise high-sensitivity imaging; (2) high spatial resolution to reveal minute biological and intracellular structures; (3) real-time imaging capabilities to capture dynamic changes during biological processes and complex surgical procedures; and (4) availability of various probe types to address different surgical requirements 76.

Research into near-infrared fluorescence probes is expanding, especially within the scope of liver cancer imaging. Current techniques primarily focus on imaging in two regions: near-infrared region I (NIR-I) and near-infrared region II (NIR-II). Each region offers unique imaging characteristics that contribute to liver cancer detection and treatment.

#### NIR-I fluorescence imaging

NIR-I spanning 700-900 nm, has unique properties that make it highly suitable for biological imaging, especially in clinical settings. NIR-I's deep tissue penetration and favorable optical characteristics make it ideal for cancer surgery navigation, molecular imaging, and analytical chemistry applications 77,78. Several NIR fluorescence probes have been designed within this range to detect specific molecular biomarkers in tissues, cells, and molecules. These probes target diverse biomarkers, including enzymes, nitro compounds, thiols, metal ions, reactive oxygen species, and peptides, and are used in both biomedical and chemical analysis 79-81. Existing NIR-I probes can be broadly classified into three categories, as shown in **Table 1**. Currently, approximately 30 NIR-I probes are clinically available 82, with FDA-approved probes such as ICG, methylene blue, and pafolacianine being the most commonly used. Among them, ICG is frequently employed in fluorescence-guided surgeries, particularly for liver cancer resection.

**Figure 7:**
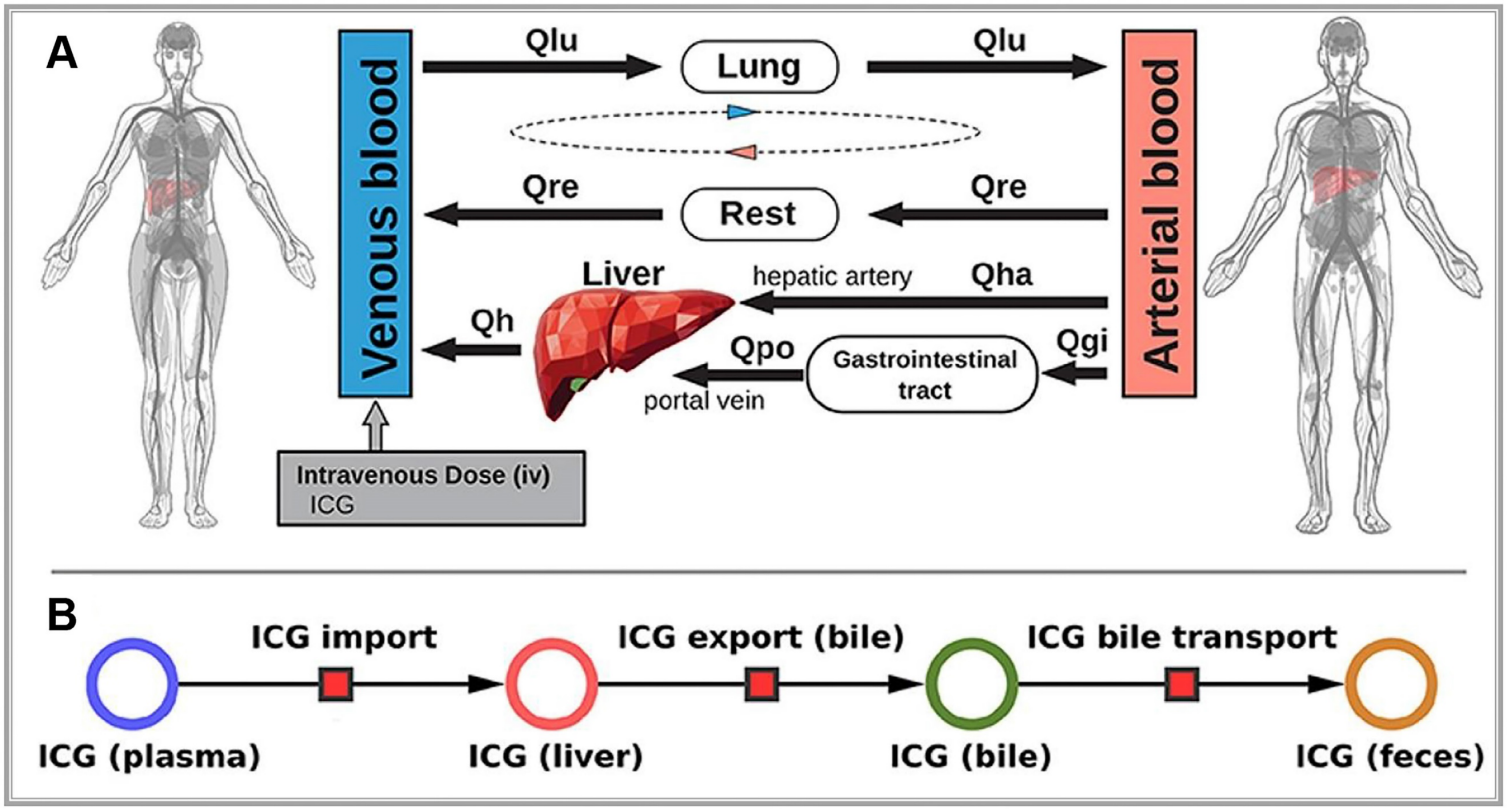
** (A)** Whole-body model: The whole-body PBPK model for ICG consists of venous blood, arterial blood, lung, liver, gastrointestinal tract, and rest compartment (accounting for organs not modeled in detail) and the systemic blood circulation connecting these compartments.** (B)** Liver model: ICG in the liver plasma compartment is taken up into the liver tissue (hepatocytes). Subsequently hepatic ICG is excreted in the bile from where it is excreted in the feces. No metabolization of ICG takes place in the liver. Reproduced with permission from 99, copyright 2021, Frontiers Media S.A.

ICG is a water-soluble dye that binds tightly to plasma proteins, such as albumin when injected intravenously. It is primarily cleared from the plasma by hepatocytes and excreted into the bile, eventually reaching the intestine (**Figure 7A**). Notably, ICG does not undergo metabolic changes during this process not participate in enterohepatic circulation (**Figure 7B**) 99. Therefore, the rate of ICG clearance from the blood to the bile duct directly serves as a direct indicator of liver reserve function 100. Specifically, the ICG 15-min blood retention rate has been well-recognized globally, particularly in the frequent use of preoperative liver functional reserve assessment 101,102. Meanwhile, when exposed to light in the 750-810 nm range, ICG emits light at around 830 nm within the NIR-I range 103. In the biological body, because of the scattering and absorption effects of hemoglobin and water molecules, light's penetration ability rapidly decreases. Hemoglobin absorbs strongly below 700 nm, while water is essentially transparent to visible and near-infrared light but strongly absorbs light above 900 nm. Thus, light in the NIR-I range (700-900 nm) achieves optimal penetration in biological tissues 104. Due to these properties, ICG fluorescence can be detected as deep as approximately 10 mm beneath the tissue surface. Accordingly, ICG has been widely used in clinical applications for over 50 years. Although rare, mild allergic reactions are occasionally reported in patients due to the iodine content in ICG during long-term use 105.

The use of ICG for NIR-I imaging in liver cancer was pioneered in 2009 by Professor Ishizawa in Japan who performed real-time NIR-I imaging during surgeries on 63 liver cancer patients to delineate tumor boundaries. In eight of these cases, fluorescence-guided imaging revealed small, previously undetected lesions 106. In 2014, Sakoda further advanced the approach by using ICG-guided NIR-I imaging to determine tumor boundaries, performing resections solely based on these imaging results with no adverse outcomes in patients 107. Recent meta-analyses have also confirmed that ICG fluorescence NIR-I imaging effectively improves clinical outcomes in liver cancer surgeries 108-110. With the widespread use of ICG NIR-I fluorescence imaging, ICG administration methods have been more meticulously studied, refining ICG dosage and timing protocols. For instance, Wakabayashi analyzed 72 relevant articles and concluded that for negative staining (detection of non-fluorescent tissues), a dose of 2.5 mg is optimal. By contrast, for positive staining (fluorescent tissue detection), a dose of 0.25 mg per patient is recommended 111. Such findings provide a theoretical basis for standardizing ICG use in clinical settings.

Furthermore, in addition to high-performance imaging probes, imaging devices are essential for achieving excellent performance in NIR-I imaging. Beyond probe technology, the development of high-performance NIR-I imaging devices has also matured, focusing currently on improving instruments by enhancing operational convenience and refining data processing capabilities. For example, the novel "Click-on" fluorescence detector developed by Oosterom 112 converts robotic surgical tools into molecular sensors, allowing surgeons to detect NIR signals under standard white light conditions. In a pig model, this technology was successfully tested for surgical applications such as vascular imaging and lymphatic mapping. Regarding data refining, Azargoshasb 113 demonstrated an innovative approach to improve fluorescence-guided surgery by tracking the real-time positions of surgical instruments. By converting positional data into dynamic digital information, they calculated the relationship between instrument position and signal-to-background ratio (SBR) to quantify the impact of SBR on fluorescence-guided surgery conducted for lesion discrimination. Results revealed that when SBR fell below 1.5, differentiating reflected excitation light from low-intensity fluorescence emission signals became difficult, increasing the risk of surgical errors. Therefore, by combining SBR quantification with kinematic scoring, this approach provides an enhanced framework for precision in fluorescence-guided surgery in clinical practice.

#### NIR-II fluorescence imaging

Compared with NIR-I imaging, fluorescent probes in the NIR-II range emit fluorescence signals in the range of approximately 1000-1700 nm 114. This spectral window is further divided into subregions, including NIR-IIa' (1000-1300 nm), NIR-IIa (1300-1400 nm), and NIR-IIb (1500-1700 nm) 77. While NIR-I imaging offers substantial improvements over visible light wavelengths, emerging research suggests that NIR-II imaging further enhances image quality in live subjects. The key advantages of NIR-II imaging are as follows 61,78: (1) Higher tissue penetration depth: NIR-II wavelengths penetrate biological tissues more effectively than NIR-I, achieving depths up to 3 cm and reducing interference from tissue scattering and absorption; (2) Strong anti-interference capability: Biological tissues produce minimal background fluorescence within the NIR-II range, resulting in higher image contrast and improved detection of fluorescence signals; and (3) Higher image resolution: NIR-II fluorescence enables detailed, high-resolution imaging at the cellular level, making it highly relevant for clinical applications.

NIR-II *in vivo* macroscopic imaging systems are particularly useful in basic research for visualizing entire organisms or specific organs in animal models, such as mice, rats, rabbits, and monkeys. In clinical applications, NIR-II imaging assists with real-time surgical navigation, intraoperative precise imaging, and macroscopic biopsy guidance 115. NIR-II microscopic systems enable detailed structural analysis at the micro-level. Additionally, the development of NIR-II fluorescence endoscopy is advancing minimally invasive laparoscopic surgeries. Currently, clinical translation of advanced medical imaging devices, including NIR-II macroscopic, microscopic, and endoscopic systems, is now a major research focus.

A wide array of NIR-II probes is currently available, including organic fluorophores, single-walled carbon nanotubes, quantum dots, and rare-earth nanoparticles 116-118. However, these probes are mostly restricted to preclinical research. Among clinically approved NIR probes, fortunately, ICG is widely used as it emits light up to 1200 nm, extending partially into the NIR-II range, thus offering a pathway for the clinical application of NIR-II fluorescence imaging 119,120. Moreover, ICG exhibits a higher quantum yield within the NIR-II range, making it a practical candidate for clinical applications in NIR-II imaging 121. Based on this, Starosolski speculated that through interactions with lipid molecules, organic media, and plasma, ICG may exhibit enhanced NIR-II signal performance, providing potential pathways for optimizing its clinical use 122.

**Figure 8:**
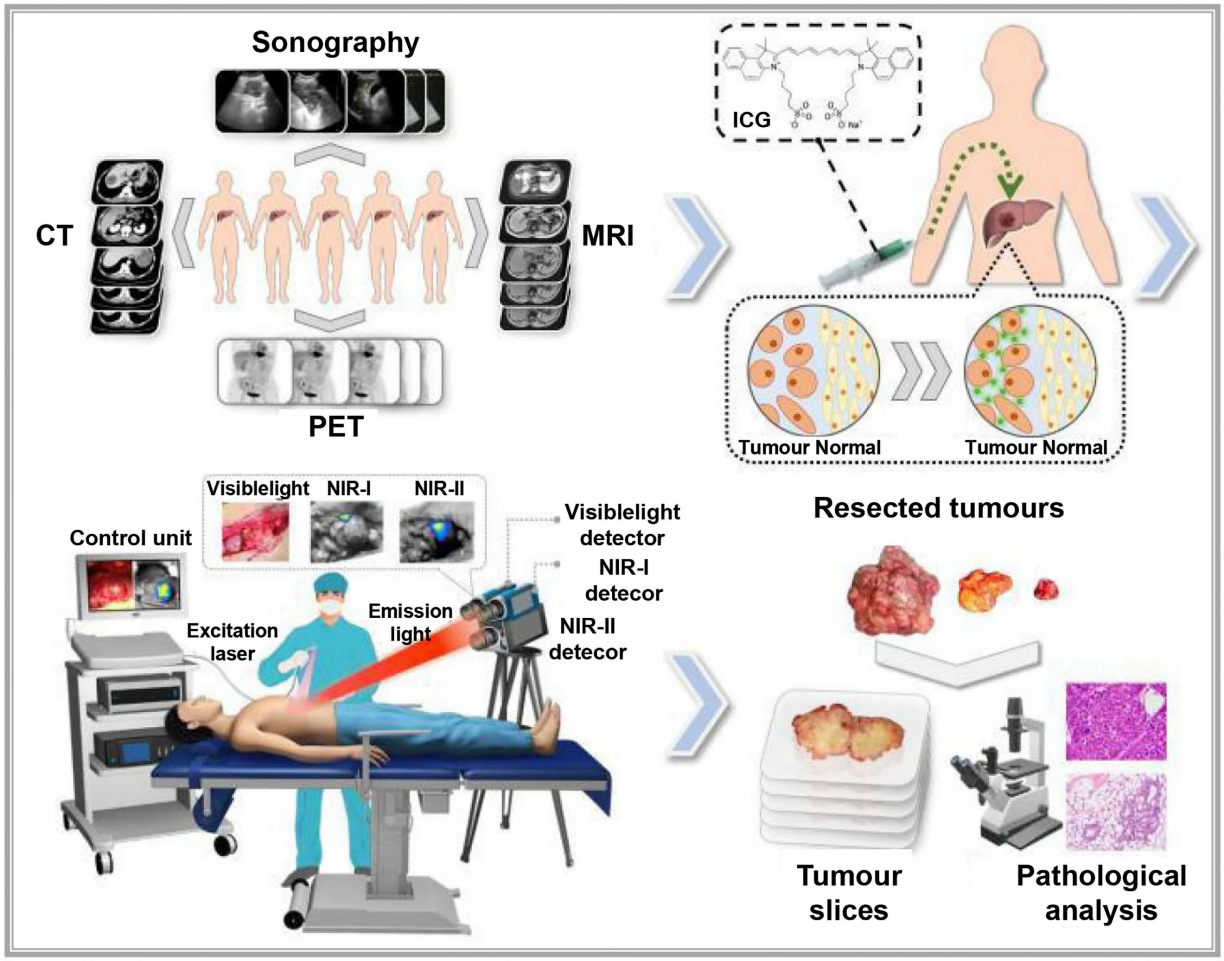
Description of the study plan and the visible and NIR-Ι/II multispectral imaging instrument for clinical applications: Patients with liver cancer were enroled in the study, and then received preoperative imaging examinations, including enhanced CT, MRI, ultrasonography and PET. Before surgery, the patients were injected with ICG intravenously at a dose of 0.5 mg/kg body weight as a routine preoperative liver function test. One to seven days later, on the day of surgery, the patients received a laparotomy. The liver surface was examined by the integrated NIR-I/II and visible multispectral imaging instrument and visible and NIR-I/II images were obtained. Tumors were resected by the guidance of ultrasonography and NIR-I imaging. During the resection, NIR-II images were also acquired. After the operation, visible and NIR-I/II images of the resected specimens were obtained. Pathological examination of the resected tissues was conducted. Reproduced with permission from 115, copyright 2022, Springer Nature.

Recent technological advances have also led to the development of more cost-effective NIR-II imaging devices by using compound semiconductor detectors made from InGaAs or HgCdTe, which capture high-sensitivity, high-contrast images in the NIR-II range 123. Multiple research teams are developing NIR-II imaging devices for practical clinical applications. For instance, a multispectral fluorescence imaging device developed by our research team, covering the spectral range of 400-1700 nm and enabling the simultaneous use of visible, NIR-I, and NIR-II imaging (**Figure 8**) 115. Using this device, they successfully conducted the first liver tumor surgery guided by multispectral fluorescence imaging, comparing tumor visibility across NIR-I and NIR-II imaging methods. This study, conducted on 23 liver cancer patients who received ICG injections, found that the tumor-to-normal-tissue ratio was higher in the NIR-II group than in the NIR-I group, both inside and outside the liver. Moreover, NIR-II imaging could detect tumor lesions that NIR-I imaging could not. The performance of NIR-II imaging in tumor identification was superior to that of NIR-I imaging. Wu constructed a portable NIR-II imaging system covering the 900-1700 nm spectral range 124. In this system, ICG was used for the first time in microsurgery navigation. The system assessed vascular patency during vessel anastomosis, arterial blood supply and venous drainage during finger replantation, and skin perforator vessels and skin flap perfusion. NIR-II imaging was found to be less affected by interfering factors than NIR-I imaging, resulting in clearer images and higher contrast, which effectively improved the accuracy of decision-making in surgical procedures.

Furthermore, in addition to hepatobiliary surgery, ICG-based NIR-II imaging has been applied in various fields. For example, Li precisely detected pancreatic cancer by using ICG-assisted NIR-I and NIR-II window fluorescence imaging techniques 125. Cao first used ICG-based NIR-II imaging for fluorescence-guided surgery in nephrectomy, with no tumor recurrence or metastasis noted in nine postoperative patients 126. In a randomized controlled trial, after conducting guided surgical resection for 33 patients with high-grade gliomas, Shi found a 100% complete resection rate in 15 patients guided by ICG-based NIR-II imaging 127. Furthermore, Shen combined *in situ* ICG NIR-II imaging with deep convolutional neural networks for pathological glioma diagnosis in real time. This approach was more sensitive than the experiential judgment of doctors (96.8% vs. 82%), which enables rapid intraoperative prediction of tumor malignancy grading and Ki-67 levels 128. This study provides robust support for the widespread clinical use of ICG's NIR-II imaging. Considering ICG is extensively used in various diagnostic and therapeutic scenarios, this technology may have broader prospects in clinical settings.

#### The new integrated model of interventional molecular imaging

In clinical diagnosis and treatment, late-stage liver cancer is often marked by subtle, invasive growth patterns that typically remain undetected until advanced stages. This progression is frequently accompanied by multiple microsatellite and metastatic foci, which can hinder early intervention efforts 129. Interventional embolization can offer a chance for surgical resection by converting otherwise inoperable cases into surgically manageable ones 130. However, even with the chance for surgical resection after interventional embolization, intraoperatively, unclear tumor tissue boundaries and hidden microlesions may not be completely and thoroughly removed, leading to a high postoperative recurrence rate. Traditional ICG-based fluorescence navigation, though helpful for identifying liver tumors, falls short in cases where embolization hinders arterial blood flow and induces cancer necrosis. These changes disrupt ICG distribution within the tumor, limiting its effectiveness for precise surgical navigation in embolization-treated liver cancer cases 131. Developing interventional molecular imaging technology, therefore, remains essential to improve therapeutic outcomes for late-stage liver cancer.

Currently, clinical research on interventional molecular imaging is limited, with the most representative work involving intraoperative segmental staining of liver cancer by injecting ICG via the ultrasound-guided portal vein or, preoperatively, through the microcatheter super-selective tumor-feeding artery under digital subtraction angiography guidance, which allows for precise fluorescence-guided surgical resection. For example, Xu successfully performed high-precision laparoscopic anatomical liver resection by injecting 5-10 mL of ICG (0.025 mg/mL) into tumor-feeding portal vein branches under intraoperative ultrasound guidance, thus primarily achieving positive staining 132. According to Li *et al.*, fluorescence-positive staining of liver segments can be achieved in laparoscopic liver cancer surgery by superselectively administering ICG through the arteries, thereby evaluating its effectiveness 133. However, the aforementioned methods, which use interventional approaches to achieve precise ICG fluorescence imaging, only address the method of ICG injection and not the drawbacks of ICG, such as its rapid metabolism as a small molecule drug, local diffusion, and fluorescence quenching. Importantly, effective approaches for implementing fluorescence navigation after liver cancer embolization are not available.

Combining interventional embolic agents with fluorescent molecular dyes is promising in addressing the downstaging of advanced liver cancer while enabling fluorescence-guided surgical diagnosis and treatment. However, developing an ideal fluorescent-labeled embolic agent requires overcoming two main challenges: achieving a uniform fluorescent dye dispersion within the embolic agent and maintaining the dye's stability for an extended period, ensuring that the fluorescence optical properties of the dye remain unaffected. To address these issues, our team developed a super-stable homogenization technique that creates a stable, uniformly mixed formulation of clinical lipiodol embolic agents with ICG, known as SHIFT&ICG. This novel formulation is being rapidly applied in the downstaging and fluorescence-guided resection of advanced liver cancer 134. In a clinical retrospective study, the SHIFT&ICG formulation prepared using this technique exhibited excellent tumor deposition efficacy and safety. During surgical resection after long-term conversion therapy for liver cancer, facilitating real-time visualization of tumor areas and boundaries, which ensures complete removal of primary lesions and tiny metastatic foci. This approach improves surgical precision while minimizing complications related to surgery, anesthesia, and postoperative recovery. SHIFT&ICG thus provides a new approach for improving the accuracy of liver cancer resection after interventional embolization therapy 131.

**Figure 9:**
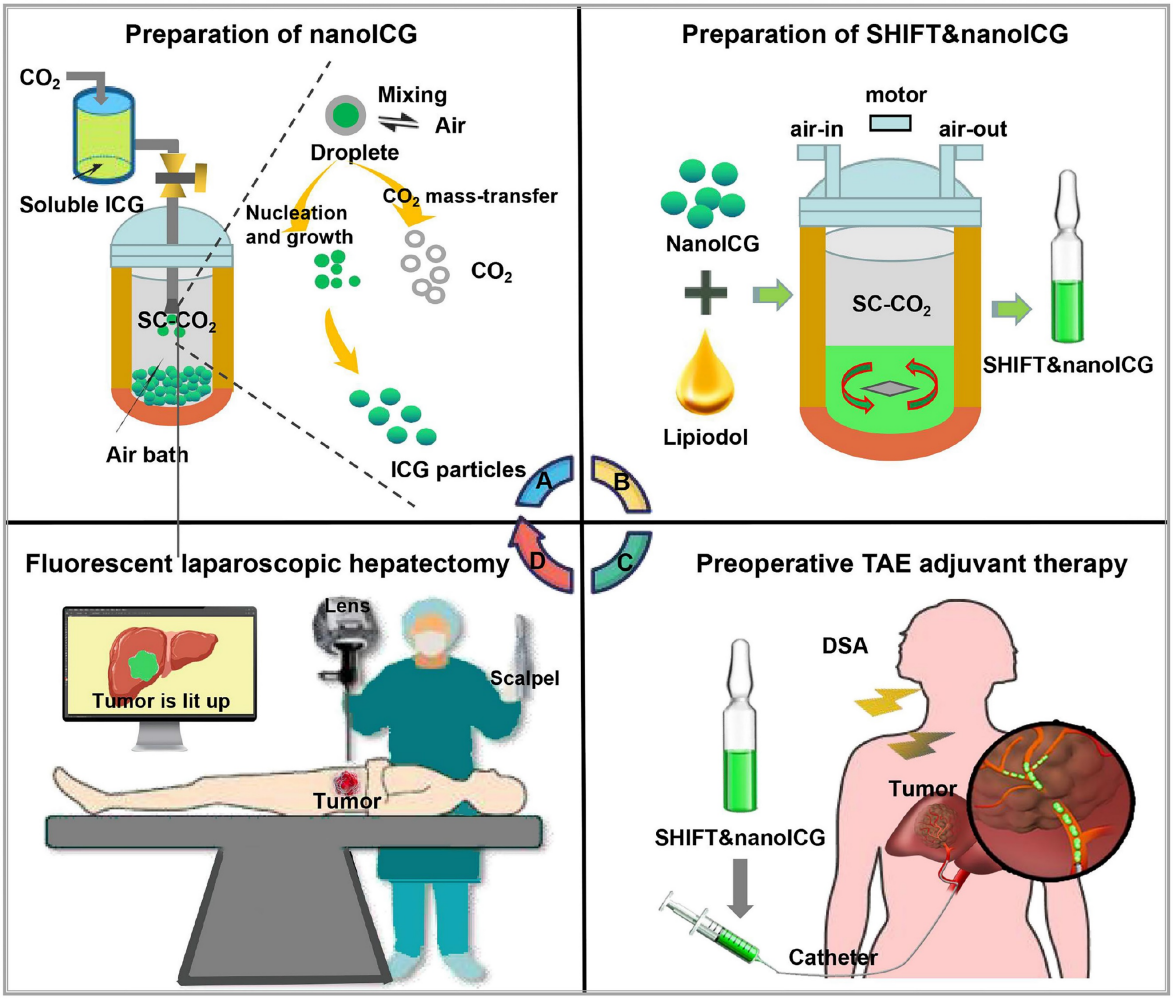
** (A)** Supercritical anti-solvent process was employed to produce carrier-free nanoICG withenhanced imaging properties and anti-photobleaching capacity. **(B)** Superstable homogeneous intermixed formulation technology (SHIFT) wasemployed to produce SHIFT&nanoICG for theranostics. **(C)** Preoperative transcatheterarterial embolization (TAE) adjuvant therapy with SHIFT&nanoICG as the embolic agent. **(D)** Thepatient received a precise laparoscopic hepatectomy under real-time fuorescence after TAE. DSA, digital subtraction angiography; SC-CO2, supercritical carbon dioxide. Reproduced with permission from 135, copyright 2022, BioMed Central.

Moreover, to further enhance fluorescence imaging performance, our team has developed a pure drug nanoparticle technology. This method nano-sizes ICG without altering its molecular structure, resulting in a formulation called SHIFT&nanoICG, which resists photobleaching and exhibits superior fluorescence imaging performance compared to conventional ICG formulations. The challenges associated with traditional ICG formulations, such as rapid decay and lower resolution, have been effectively addressed by SHIFT&nanoICG 130, which has been validated through clinical experiments. This formulation can enable accurate fluorescence navigation for microsatellite lesions that often go undetected in preoperative imaging 135. With its significant translational potential, SHIFT&nanoICG is set to redefine interventional embolization techniques for fluorescence-guided liver cancer surgery, paving the way for a more effective surgical model, the research process is shown in **Figure 9A-D.**

## Summary and prospect

The future development of NIR imaging technology in precision medicine promises to advance individualized and customized surgical solutions to patients' specific needs. For example, by integrating fluorescence navigation with other imaging modalities, clinicians may achieve enhanced detection of liver cancer and its metastatic foci, including improved assessment of tumor depth 136. Although optical molecular imaging, primarily NIR-II imaging, has been tested in various disease models, clinical research remains scarce, with few multicenter, large-sample studies providing prospective data. Simultaneously, the optimization and miniaturization of detection devices will be essential to support broader applications in surgical practice.

Looking ahead, optical molecular imaging is poised to play an increasingly significant role in liver cancer treatment. On one hand, with ongoing advancements, the technology's resolution and sensitivity will improve, allowing medical professionals to more accurately locate and identify tiny tumor lesions. On the other hand, incorporating machine learning and artificial intelligence will make molecular imaging more intelligent, supporting surgical decision-making, reducing surgical errors, and improving the success and accuracy of surgical procedures. Additionally, this technology will also be vital in postoperative follow-up, enabling clinicians to monitor recovery, track tumor recurrence, and adapt treatment plans promptly, thereby improving patients' overall survival rates and quality of life.

In biomedical research and clinical practice, any single detection method is associated with limitations. Integrating fluorescence imaging with other advanced diagnostic imaging methods or other therapeutic approaches, such as targeted therapy, and immunotherapy, is promising for further enhancing the therapeutic outcomes of liver cancer surgery. Through comprehensive treatment strategies, fluorescence imaging technology may offer more personalized and precise treatment plans for liver cancer patients 137. Although optical molecular imaging shows potential in clinical trials, transitioning these findings into routine practice presents challenges. This will require interdisciplinary collaboration, with involvement from physicians, biologists, and engineers, alongside rigorous clinical trials and regulatory approval processes. Before further clinical development, several factors must be addressed for successful clinical translation: (1) the combination of imaging agents with carriers or ligands may alter the pharmacokinetics and biodistribution of fluorescent dyes, potentially introducing new side effects; (2) mitigating the toxicity of complex probe structures is critical, especially given risks such as cardiotoxicity, renal and hepatic impairment, and neurotoxicity. Modifying the preparation techniques of imaging probes, optimizing the materials used, and selecting appropriate administration routes may provide references for addressing these issues. Developing imaging probes through carrier-free methods, such as Liu's pure drug nanotechnology, which avoids organic solvents and toxic chemicals, could help tackle these issues. Additionally, choosing clinically approved drugs or their components as raw materials for optical probes or optimizing administration routes may minimize side effects, simplify the manufacturing process, and expedite clinical application.

In conclusion, optical molecular imaging offers a promising future in precise liver cancer surgical navigation. However, realizing its full potential will require ongoing innovation and interdisciplinary collaboration. With continued advancements and extensive clinical research, fluorescence imaging technology centered on optical molecular imaging is expected to become integral to liver cancer treatment, supporting more effective, personalized, and life-enhancing outcomes.

## Figures and Tables

**Table 1:** The classification of common NIR-I probe

Category	Chemical construction	Ref.
Small molecule fluorophore	Cyanines	83
	Porphyrin-based	84
	Mental complexes	85
	Xanthene dyes	86, 87
	Squaraine	88, 89
	Phenothiazine-based	90
	Bodipy	91, 92
	Dicyanomethylene-4H-pyran	93
Synthetic nanoparticles	Single walled carbon nanotubes	94
	Quantum dots	95
	Rare earth nanomaterials	96
Biologics	Near-infrared fluorescent proteins	97
	Phytochrome	98
